# A Case of Spontaneous Splenic Rupture After a Hemicolectomy on Anticoagulation

**DOI:** 10.7759/cureus.67512

**Published:** 2024-08-22

**Authors:** William B Bowers, Landry K Umbu, Megan Contreras, Joshua K Phillips

**Affiliations:** 1 General Surgery, American University of Antigua, Coolidge, ATG; 2 General Surgery, Western Reserve Health Education, Warren, USA; 3 General Surgery, Southwoods Physician Services, Boardman, USA

**Keywords:** appendiceal adenocarcinoma, appendiceal cancers, total splenectomy, goblet cell adenocarcinoma, anticoagulation, hemicolectomy, atraumatic splenic rupture, spontaneous splenic rupture

## Abstract

Spontaneous or atraumatic splenic rupture (ASR) is a rare but life-threatening condition that requires swift recognition and intervention. We report the case of a 66-year-old female with a history of hypothyroidism, appendiceal goblet cell adenocarcinoma, and new-onset atrial fibrillation (Afib) requiring anticoagulation. She initially presented with right upper quadrant abdominal pain. She had previously undergone an appendectomy followed by a right hemicolectomy to achieve clear surgical margins after the appendiceal carcinoma diagnosis. In the post-anesthesia care unit, she developed Afib and was started on therapeutic anticoagulation. Cardiac catheterization later revealed three-vessel coronary artery disease, prompting a transition from heparin to apixaban. Three days later, the patient suddenly experienced left shoulder pain and was found to be diaphoretic and hypotensive. Three days post-catheterization, the patient developed sudden left shoulder pain, along with diaphoresis and hypotension. An initial concern for post-catheterization myocardial infarction was ruled out. A subsequent CT of the abdomen and pelvis revealed a large splenic hematoma with rupture and hemoperitoneum, necessitating emergent open splenectomy. Post-operatively, the patient required intensive care monitoring and transfusion support before being discharged to a long-term acute care facility.

ASR is typically associated with identifiable pathological conditions; however, this case highlights the complexity of multifactorial etiologies. It emphasizes the need to consider ASR in patients presenting with sudden left shoulder pain and hemodynamic instability, particularly when anticoagulation therapy or recent abdominal surgery are factors. This case underscores the importance of a high index of suspicion and timely intervention to prevent fatal outcomes. Further research is warranted to explore the relationship between anticoagulation therapy and ASR.

## Introduction

Spontaneous or atraumatic splenic rupture (ASR) is rare. The incidence is not well-defined, though one study suggested that approximately 3.2% of splenic rupture cases are spontaneous, with a mortality rate of around 12% [1]. This case report presents a 66-year-old female with a finding of appendiceal goblet cell adenocarcinoma, who underwent a right hemicolectomy to ensure clear surgical margins. During her post-operative course, she developed atrial fibrillation (Afib) and began therapeutic anticoagulation. Cardiac catheterization, performed for newly identified Afib complicated by congestive heart failure, revealed three-vessel coronary artery disease. Three days after the cardiac catheterization and transition to apixaban, she developed left shoulder pain, diaphoresis, and hypotension. A CT scan revealed a large splenic hematoma with rupture and hemoperitoneum, leading to an emergent open splenectomy.

The cause of ASR is often pathological, but iatrogenic causes have been demonstrated. Severe splenomegaly is the most common cause with etiologies including infections (e.g., mononucleosis), leukemia, inflammatory causes, medications (e.g., anticoagulation), and iatrogenic causes [2-5]. The patient's complex medical history, including neoplasm, inflammation from appendicitis and intra-abdominal abscess, Afib with anticoagulation, and hemicolectomy, likely contributed to the splenic rupture. Definitive etiology is challenging to ascertain due to the complexity of her case.

## Case presentation

**Figure 1:**
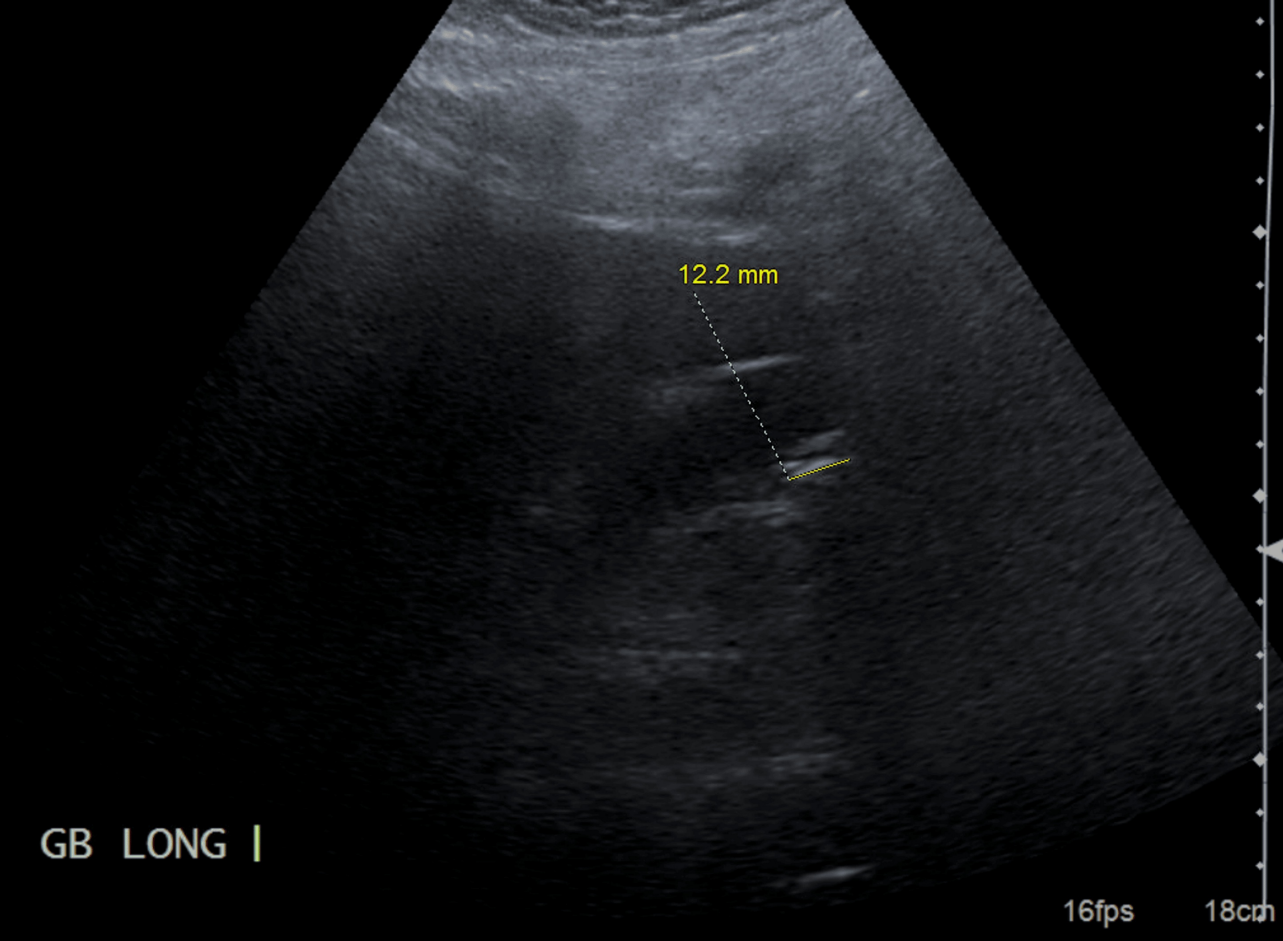
Ultrasound of the right upper quadrant measuring a normal gallbladder length and without findings of gallbladder wall thickening or pericholecystic fluid. The yellow line indicates the length of the gallbladder.

The patient, a 66-year-old female tobacco smoker with a medical history of hypothyroidism and a past surgical history of a hysterectomy, presented to the emergency department with a three-day history of worsening right upper quadrant abdominal pain. She was hemodynamically stable upon arrival. Her abdomen was tender in the right upper quadrant without signs of peritonitis. Laboratory results showed leukocytosis (22,300/uL; reference range for white blood cell (WBC) count: 4,000 to 11,000 cells/uL), mild lactate elevation (3.1 mmol/L; reference range for lactate: 0.5 to 2.2 mmol/L), elevated total bilirubin (1.6 mg/dL; reference range for total bilirubin: 0.1 to 1.2 mg/dL), and normal alkaline phosphatase (78 U/L; reference range for alkaline phosphatase: 44 to 147 U/L). An abdominal right upper quadrant ultrasound (Figure 1) showed cholelithiasis with a positive Murphy sign but no wall thickening or fluid collection. Surgery was consulted for symptomatic cholelithiasis, and a laparoscopic cholecystectomy was planned.

Upon entering the abdomen, there was purulence throughout. Extensive adhesiolysis visualized the gallbladder, and against clinical suspicion, it was unlikely to be the source. Systematic inspection of the abdomen and bowel identified an acutely perforated appendix, which was isolated, stapled, and sent for pathology. The patient tolerated the procedure well.

Post-operatively, the patient developed new-onset Afib with a rapid ventricular response (RVR). An echocardiogram showed dilated cardiomyopathy and heart failure. An unfractionated heparin drip was started, and cardiology planned for cardiac catheterization when she was stable on hospital day six. On hospital day 10, pathology confirmed appendiceal goblet cell adenocarcinoma; unfortunately, histopathological images were not obtainable for this report. Surgery was planned to achieve clear margins.

A right hemicolectomy with side-to-side ileocolic anastomosis was performed on hospital day 12. The patient tolerated the procedure well. Cleared by surgery, on hospital day 22, the patient went for cardiac catheterization for three-vessel coronary artery disease. She was found to have heart failure (EF of 19%). After cardiac catheterization, the patient transitioned from heparin to apixaban. 

**Figure 2:**
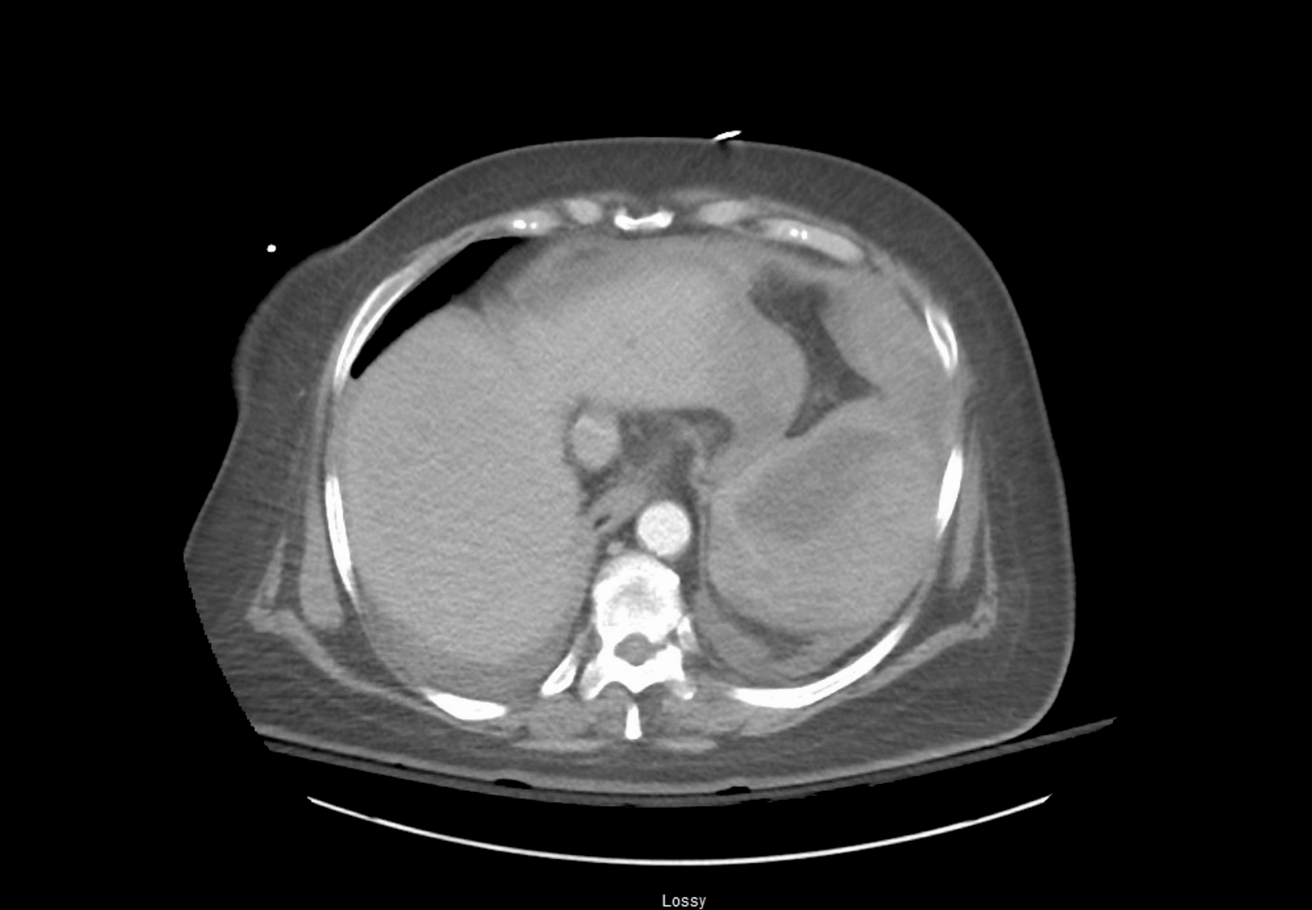
Axial view of contrast-enhanced computed tomography (CT) of the abdomen and pelvis illustrating a large splenic hematoma with rupture and compression of the normal splenic tissue.

**Figure 3:**
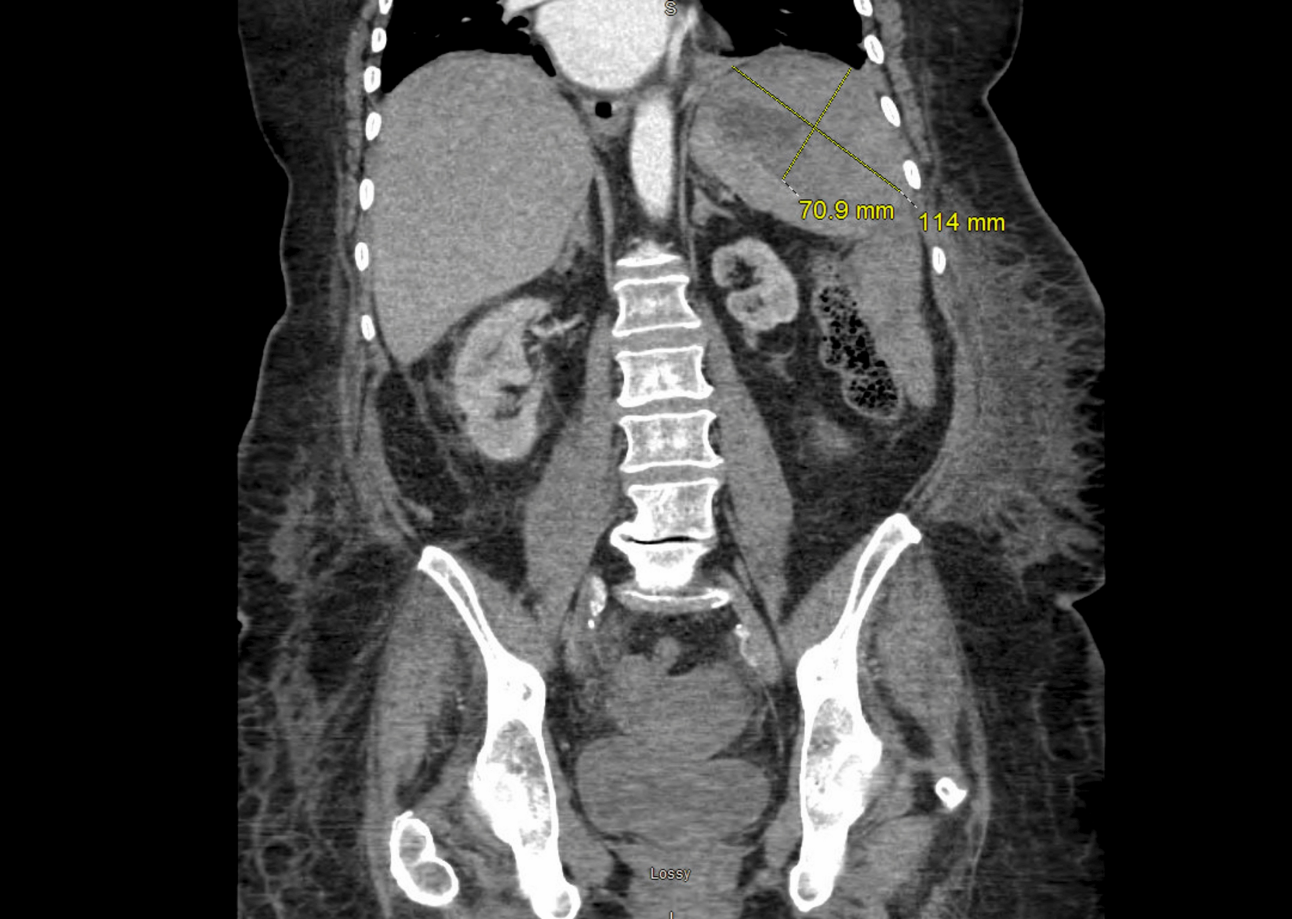
Sagittal view of contrast-enhanced computed tomography (CT) of the abdomen and pelvis illustrating large splenic hematoma measuring 11 x 7 cm, with compression of the normal splenic tissue. Yellow lines measure the length and width of the splenic hematoma.

Three days after her cardiac catheterization and 12 days after hemicolectomy, the patient began to experience acute left upper quadrant pain with radiation to her left shoulder. She was found to be diaphoretic and hypotensive. Initial concern for a post-catheterization myocardial infarction was worked up and ruled out. A CT abdomen and pelvis (Figures 2, 3) demonstrated a large splenic hematoma with rupture and hemoperitoneum. The patient was taken for emergent open splenectomy. The abdomen was explored with findings of over one liter of hemoperitoneum and a ruptured spleen. The spleen was medialized by releasing the splenophrenic and splenocolic ligaments, and the splenic hilum was stabilized. Hemostasis was achieved, and the abdomen was carefully examined for other sources of bleeding. The patient required two units of packed red blood cells (pRBCs) and one unit of fresh frozen plasma. Post-operatively, she was hemodynamically stable.

fThe patient was transferred to the ICU for monitoring with serial hemoglobin and hematocrit measurements. She received an additional three units of pRBCs (totaling five). Necessary post-splenectomy vaccinations were administered on hospital day 30, and she was discharged to a long-term acute care facility on hospital day 31.

## Discussion

ASR is a rare and life-threatening condition with various identified causes. Most ASR cases are linked to pathological conditions such as infection, neoplasm, inflammatory processes, medications, and mechanical causes like pregnancy and iatrogenic procedures. Common infectious causes include mononucleosis, malaria, and HIV. According to a systematic review of over 845 cases, ASR is idiopathic in about 7% of cases and more often associated with pathology [1]. A pathological spleen, due to its altered state and fragility, is more likely to rupture due to even minor stresses [6,7]. Iatrogenic causes are also noted, with procedures such as endoscopy being notable culprits.

Hematological causes of ASR, such as lymphoma and leukemia, are documented, but there is limited association between solid neoplasms and ASR, except for metastatic splenic nodules from other sources, often lung cancer [8] and local invasion such as pancreatic carcinoma [9]. This patient had goblet cell appendiceal adenocarcinoma, which can disseminate within the abdomen [10]. Treatment typically involves appendectomy followed by right hemicolectomy. Some studies suggest additional procedures, but this patient had already undergone a hysterectomy [10]. No established link exists between goblet cell adenocarcinoma of the appendix and dissemination to the spleen.

Certain medications are associated with ASR, including anticoagulants, thrombolytics, and granulocyte colony-stimulating factors [1]. Dual antiplatelet therapy with heparin and ticagrelor has been implicated in ASR [2]. Other cases report ASR related to ticlopidine [11] and chronic or acute apixaban therapy [3,4]. Heparin therapy has been less frequently reported as a cause [12]. In this case, the patient was anticoagulated for Afib with intravenous heparin and later transitioned to apixaban, potentially contributing to her ASR.

Iatrogenic triggers for ASR include colonoscopy, hemodialysis, laparoscopic procedures, and lower left-sided thoracic procedures [5,13-15]. Although the patient's recent laparotomy and right hemicolectomy might be a plausible cause, ASR typically presents within hours to days of the procedure [13,14]. A small injury during the procedure could be exacerbated by transitioning to apixaban, but this is challenging to assess without pre-symptom CT imaging.

Inflammation and infection are common ASR causes, but unlikely in this case given the absence of identified infections despite prolonged leukocytosis. The leukocytosis might have been due to the solid tumor or appendicitis.

Management of traumatic splenic rupture usually involves total splenectomy. For ASR, management varies but often includes total splenectomy [1]. Other options include organ-preserving surgery like splenorrhaphy or non-surgical treatments like splenic artery embolization [1]. Conservative management is often less successful (13%) [1]. Given the patient’s age and anticoagulation therapy, splenectomy was necessary. The patient was hemodynamically unstable, making splenectomy unavoidable.

## Conclusions

The cause of ASR can be multifactorial. This patient was found to have goblet cell adenocarcinoma causing appendicitis with rupture, atrial fibrillation with anticoagulation therapy, and hemicolectomy. Inflammation and anticoagulative and iatrogenic causes may have played a role in the rupture of this patient's spleen. Analysis of definitive etiology is limited due to the complexity of care given and multiple factors at play. Research is limited to the rarity of ASR and goblet cell adenocarcinoma in the appendix. The infrequency of both has left only case reports and a few systematic reviews of such reports. The limitation in the current literature should push clinicians to consider ASR when presented with new left shoulder pain and hemodynamic instability in relation to anticoagulation and/or procedures. 
